# Supplementary material for: Artemisinin exposure at the ring or trophozoite stage impacts Plasmodium falciparum sexual conversion differently
Source: eLife. 2020 Oct 21;9:e60058. doi: 10.7554/eLife.60058 (PMC7577739; doi:10.7554/eLife.60058)
Supplement: Transparent reporting form [file elife-60058-transrepform.pdf]

1st Floor
24 Hills Road
Cambridge CB2 1JP, UK

P 01223 855340
W elifesciences.org
T @elife

eLife's transparent reporting form

We encourage authors to provide detailed information *within their submission* to facilitate the interpretation and replication of experiments. Authors can upload supporting documentation to indicate the use of appropriate reporting guidelines for health-related research (see EQUATOR Network), life science research (see the BioSharing Information Resource), or the ARRIVE guidelines for reporting work involving animal research. Where applicable, authors should refer to any relevant reporting standards documents in this form.

If you have any questions, please consult our Journal Policies and/or contact us: editorial@elifesciences.org.

Sample-size estimation

- You should state whether an appropriate sample size was computed when the study was being designed
- You should state the statistical method of sample size computation and any required assumptions
- If no explicit power analysis was used, you should describe how you decided what sample (replicate) size (number) to use

Please outline where this information can be found within the submission (e.g., sections or figure legends), or explain why this information doesn't apply to your submission:

This manuscript does not include any population-based or epidemiological research, so we did not perform sample size calculations. For our lab-based experiments, we selected the number of independent biological replicates based on feasibility and variability between replicates. When differences (or absence of differences) between conditions were obvious, such that the same patterns were consistently observed in all replicates and also in similar experiments under slightly different conditions (which could not be counted as replicates), and results were consistent with analyses with other parasite lines, we performed a minimum of 2 independent biological replicates (typically 3). However, the main conclusions of the manuscript are based on experiments with 4 independent biological replicates and validation of the results with different parasite lines.

Replicates

- You should report how often each experiment was performed
- You should include a definition of biological versus technical replication
- The data obtained should be provided and sufficient information should be provided to indicate the number of independent biological and/or technical replicates
- If you encountered any outliers, you should describe how these were handled
- Criteria for exclusion/inclusion of data should be clearly stated
- High-throughput sequence data should be uploaded before submission, with a private link for reviewers provided (these are available from both GEO and ArrayExpress)

Please outline where this information can be found within the submission (e.g., sections or figure legends), or explain why this information doesn't apply to your submission:

The number of independent biological replicates is indicated in the figure legends. Individual data points, in addition to average and SEM, are shown in the figures, when possible. We define independent biological replicates as experiments performed with samples collected from different parasite cultures, on separate days.

In addition, for transcriptional analysis by RT-qPCR we used technical replicates, which in this case means analysis of the same cDNA samples in multiple wells of a qPCR plate. This is indicated in the Methods section (under the heading "Transcriptional analysis").

No outliers were excluded. All results obtained after the optimization of the assays are included in the manuscript.

eLife Sciences Publications, Ltd is a limited liability non-profit non-stock corporation incorporated in the State of Delaware, USA, with company number 5030732, and is registered in the UK with company number FC030576 and branch number BR015634 at the address 1st Floor, 24 Hills Road, Cambridge CB2 1JP | August 2014

Statistical reporting

- Statistical analysis methods should be described and justified
- Raw data should be presented in figures whenever informative to do so (typically when N per group is less than 10)
- For each experiment, you should identify the statistical tests used, exact values of N, definitions of center, methods of multiple test correction, and dispersion and precision measures (e.g., mean, median, SD, SEM, confidence intervals; and, for the major substantive results, a measure of effect size (e.g., Pearson's r, Cohen's d)
- Report exact p-values wherever possible alongside the summary statistics and 95% confidence intervals. These should be reported for all key questions and not only when the p-value is less than 0.05.

Please outline where this information can be found within the submission (e.g., sections or figure legends), or explain why this information doesn't apply to your submission:

Statistical methods are described and justified in the Methods section, under the "Determination of sexual conversion rates" and "Production of mature gametocytes and mosquito feeding" headings. Individual data points are shown in the figures whenever possible, and exact p-values are provided. Data are typically provided as average and SEM.

We chose to perform statistical analysis only for sexual conversion rates, which is the main outcome of this study and the more stable parameter (e.g., it is not affected by unavoidable small variation in initial parasitemia between experiments), for mosquito infection experiments and for transcriptional analyses. There is an intense debate in the community about the appropriateness of using statistical analysis for studies that intrinsically involve only a small number of replicates. We considered appropriate to focus mainly in the magnitude of the differences reported, but still provide p-values for the main parameters.

(For large datasets, or papers with a very large number of statistical tests, you may upload a single table file with tests, Ns, etc., with reference to sections in the manuscript.)

Group allocation

- Indicate how samples were allocated into experimental groups (in the case of clinical studies, please specify allocation to treatment method); if randomization was used, please also state if restricted randomization was applied
- Indicate if masking was used during group allocation, data collection and/or data analysis

Please outline where this information can be found within the submission (e.g., sections or figure legends), or explain why this information doesn't apply to your submission:

N.A.

Additional data files ("source data")

- We encourage you to upload relevant additional data files, such as numerical data that are represented as a graph in a figure, or as a summary table

- Where provided, these should be in the most useful format, and they can be uploaded as “Source data” files linked to a main figure or table
- Include model definition files including the full list of parameters used
- Include code used for data analysis (e.g., R, MatLab)
- Avoid stating that data files are “available upon request”

Please indicate the figures or tables for which source data files have been provided:

All information is provided in the Main manuscript file or the Supplementary information file. We consider that providing numerical values for all data in the bar charts would be redundant and would not add any relevant information.
